# 

**Published:** 1898-03

**Authors:** 


### CONTENTS.

PROCEEDINGS.

Ohio State Dental Society .......... 101

COMMUNICATIONS.

Instrument Nomenclature with Reference to Instrumentation .......... 125
An Interview .......... 135
Salivary Calculi .......... 138

SELECTIONS.

An Attempt Likely to Fail .......... 137
Surgeon-General Sternberg .......... 143
A New Local Anesthetic .......... 144
A New Element in the Blood .......... 145
Disinfection of the Mouth .......... 145

NOTICES.

American Dental Society of Europe .......... 145
Illinois State Dental Society .......... 146
Next Meeting of the American Medical Association, June 7th to 10th, 1898 .......... 146

EDITORIAL

The Odontographic Society of Chicago .......... 147
Tri-State Meeting .......... 148

OBITUARY.

Mr. Ernest Hart .......... 149
In Memory of Dr. W. B. Chambers .......... 150

